# The unexpected role of RagC in ketogenic diet-derived BHB sensing

**DOI:** 10.1093/procel/pwag029

**Published:** 2026-05-03

**Authors:** Jian Fu, Xiaoqian Fu, Wenkai Ren

**Affiliations:** The First Affiliated Hospital of Guangxi Medical University, Nanning 530021, China; State Key Laboratory of Swine and Poultry Breeding Industry, Guangdong Laboratory of Lingnan Modern Agriculture, College of Animal Science, South China Agricultural University, Guangzhou 510642, China; The First Affiliated Hospital of Guangxi Medical University, Nanning 530021, China; The First Affiliated Hospital of Guangxi Medical University, Nanning 530021, China; State Key Laboratory of Swine and Poultry Breeding Industry, Guangdong Laboratory of Lingnan Modern Agriculture, College of Animal Science, South China Agricultural University, Guangzhou 510642, China; Yuelushan Laboratory, Changsha 410128, China

The mechanistic target of rapamycin complex 1 (mTORC1) has long been viewed as a central hub that integrates nutrient and energy signals to control cell growth, with its dysregulation implicated in metabolic syndromes, neurodegeneration, and cancer (Gan et al., 2024; Kim and Guan, 2019). This paradigm, largely built on studies of amino acid sensing through non-covalent interactions with adaptor proteins, such as sestrin2, CASTOR1, and secretion-associated Ras-related GTPase 1B (SAR1B), posits that mTORC1 activation is primarily governed by direct binding events (Chantranupong et al., 2016; Chen et al., 2021; Wolfson et al., 2016). However, in a recent study published in *Protein* & *Cell*, Dr. Deng’s group demonstrate that the ketone body β-hydroxybutyrate (BHB), a major metabolite produced during ketogenic diet (KD), suppresses mTORC1 activity through covalent modification of RagC via lysine β-hydroxybutyrylation (Kbhb) at residue K349, thereby inhibiting colorectal cancer (CRC) growth (Fig. 1) (Wang et al., 2026).

**Figure 1.**
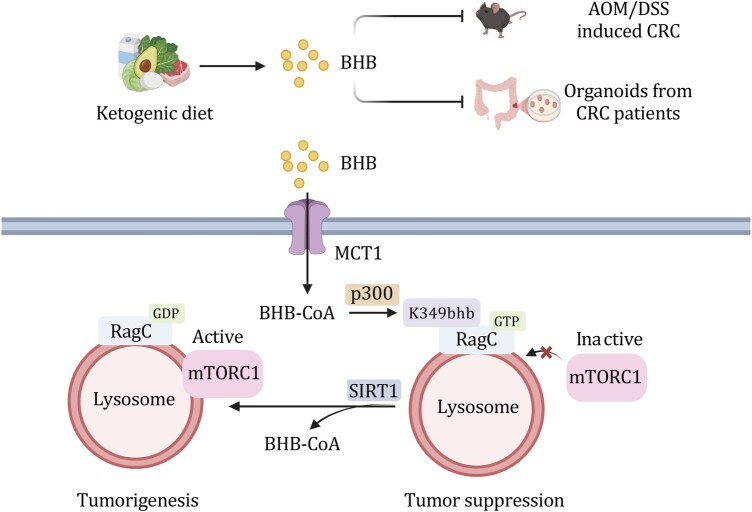
**RagC-mediated sensing of BHB suppresses mTORC1 signaling and CRC**. BHB induces Kbhb of RagC at residue K349, suppressing mTORC1 signaling. p300 catalyzes this modification, which is reversed by SIRT1, leading to impaired mTORC1 activity and reduced colorectal cancer growth. This dynamic modification thus links ketone metabolism to tumor growth regulation.

Using proteomic and metabolic approaches, the authors identify RagC as the primary target of BHB-induced Kbhb among Rag GTPases, with modification occurring specifically at K349 (K348 in mice). Notably, this modification is exclusive to D-BHB and is not induced by its enantiomer, L-BHB, whereas a recent study showed that BHB modifies aldolase B to indirectly inhibit mTORC1 in liver cancer models without distinguishing between its D- and L-forms (Qin et al., 2024). It should be noted that the KD-derived BHB reaches only 2–3 mmol/L in circulation, whereas many previous studies employed supraphysiological concentrations (5–50 mmol/L) (Dmitrieva-Posocco et al., 2022; Han et al., 2025; Huang et al., 2021; Li et al., 2022). To address this critical gap, the authors deliberately constrained BHB to less than 1 mmol/L throughout their experiments, ensuring physiological relevance. Certainly, this design choice significantly elevates the translational relevance of their findings, bridging the divide between experimental observation and potential clinical application.

Interestingly, only p300, and not the other acetyltransferases tested, promotes RagC Kbhb, consistent with a previous study showing that p300 is a key writer of Kbhb (Huang et al., 2021). Conversely, screening of deacetylases revealed that SIRT1, but not SIRT2-5, removes this modification. Notably, SIRT3 has been reported to erase Kbhb on histones (Zhang et al., 2019), suggesting that SIRT family members may function as Kbhb erasers across different cellular compartments. These observations together suggest that RagC Kbhb is dynamically regulated by the p300-SIRT1 axis, representing a new layer of nutrient-sensitive signaling. It is enigmatic that BHB treatment does not alter the enzymatic activity of p300 or SIRT1. It should be noted that BHB availability likely drives modification through substrate (BHB-CoA) availability rather than enzyme activation (Huang et al., 2021). This substrate-driven model challenges assumptions of canonical enzymatic regulation and highlights the primacy of metabolite availability in shaping cellular responses. However, whether BHB-CoA itself serves as the direct modifying agent or its accumulation indirectly alters pathway flux remains unresolved, a question that demands systematic exploration to disentangle substrate versus enzyme contributions in ketone body biology.

The functional consequences of RagC K349bhb were then elucidated through structural and cellular analyses. Molecular docking revealed that unmodified RagC-K349 forms a stabilizing hydrogen bond with regulatory-associated protein of mTOR (Raptor)-K1221, whereas K349bhb disrupts this interaction despite an expanded binding interface. This disruption prevents mTORC1 recruitment to the lysosomal surface, a process known to require direct RagC-Raptor interaction (Rogala et al., 2019; Sancak et al., 2008). Accordingly, BHB inhibits mTORC1 signaling (p-S6K, p-S6) and suppresses CRC cell viability, organoid growth, and tumor progression in cell-derived xenograft (CDX) and azoxymethane (AOM)/dextran sodium sulfate (DSS) mouse models. Importantly, these effects are completely abrogated in RagC-K349R knockin cells and mice, where the lysine is replaced by arginine to preclude Kbhb modification. The specificity of this mechanism is further underscored by the stereochemical selectivity for D-BHB over L-BHB. Together, these findings establish that RagC-K349bhb is both necessary and sufficient for BHB-mediated mTORC1 suppression.

Notably, the overexpression of RagC-K349R in human CRC organoids abrogates BHB-mediated suppression of proliferation and restores mTORC1 activity. In addition, analysis of CRC patient samples reveals a significant inverse correlation between RagC-K349bhb levels and both mTORC1 activity (p-S6) and tumor markers (CEA, CA199). Patients with lower tumor tissue BHB levels exhibit larger tumors and elevated CA199. These data highlight the clinical relevance of the RagC-K349bhb axis in human CRC. However, the role of BHB in CRC is more complex than simple inhibition. Recent evidence indicates that BHB may paradoxically facilitate CRC progression by acting as an alternative energy source, particularly in cancer cells harboring mutations in critical genes such as *APC*, *KRAS*, and *TP53* (Rashidi et al., 2026). It should be noted, however, that this study does not elucidate whether the RagC-K349bhb pathway is affected by these genetic mutations, nor does it explore how K349bhb affects RagC nucleotide binding or its interaction with the folliculin (FLCN) complex (Tsun et al., 2013). Future studies are required to determine whether this modification induces structural rearrangements within RagC’s nucleotide-binding domains. Furthermore, stratifying patients according to their genetic profiles (*APC*, *KRAS*, *TP53* status) may identify those most likely to benefit from KD or BHB supplementation. Exploring the therapeutic potential of combining BHB with targeted therapies or immune checkpoint blockade will be crucial for translating these preclinical findings into combination strategies.

In summary, a recent study by Dr. Deng’s group systematically reveals a previously unrecognized axis connecting nutrient metabolism and tumor signaling regulation. This study elucidated the molecular mechanism by which BHB generated from a ketogenic diet inhibits colorectal cancer growth via RagC-K349bhb‑mediated suppression of the mTORC1 pathway, suggesting that the dynamic RagC‑K349bhb modification may serve as a novel potential target for colorectal cancer intervention.

## Data Availability

Not applicable.
